# Supplementary material for: Quantifying the risk of spillover reduction programs for human health
Source: PLoS Comput Biol. 2024 Aug 15;20(8):e1012358. doi: 10.1371/journal.pcbi.1012358 (PMC11349207; doi:10.1371/journal.pcbi.1012358)
Supplement: S1 Text — This file includes complete derivations of analytical results, descriptions of supporting numerical analyses, descriptions of supporting data, and supporting figures. (PDF) [file pcbi.1012358.s001.pdf]

Supporting information: Quantifying the risk of spillover reduction programs for human health

Scott L. Nuismer[1,*]
Andrew J. Basinski[2]
Courtney L. Schreiner[3]
Evan A. Eskew[2]
Elisabeth Fichet-Calvet[4]
Christopher H. Remien[5]

July 2024

1. Department of Biological Sciences, University of Idaho, Moscow, Idaho 83844

2. Institute for Interdisciplinary Data Sciences, University of Idaho, Moscow, Idaho 83844

3. Department of Ecology and Evolutionary Biology, University of Tennessee, Knoxville, Tennessee 37996

4. Bernhard Nocht Institute for Tropical Medicine, Hamburg, Germany

5. Department of Mathematics and Statistical Science, University of Idaho, Moscow, Idaho 83844

* Corresponding author; e-mail: snuismer@uidaho.edu.

Supporting Information

Steady state solutions

At steady state, the system of partial differential equations (1) reduces to the following system of ordinary differential equations:

$$\frac{dS}{da} = -\lambda S + \omega R - \delta S \tag{S1a}$$
$$\frac{dI}{da} = \lambda S - \gamma I - \delta I - \mu I \tag{S1b}$$
$$\frac{dM}{da} = \mu I - \gamma M - \delta M - vM \tag{S1c}$$
$$\frac{dR}{da} = \gamma(I + M) - \omega R - \delta R \tag{S1d}$$

with initial condition $S(0) = b$, $I(0) = 0$, $M(0) = 0$, and $R(0) = 0$

If all parameters are independent of age, the abundance of each class can be found by solving the following system of equations:

$$0 = b - \lambda S + \omega R - \delta S \tag{S2a}$$
$$0 = \lambda S - \gamma I - \delta I - \mu I \tag{S2b}$$
$$0 = \mu I - \gamma M - \delta M - vM \tag{S2c}$$
$$0 = \gamma(I + M) - \omega R - \delta R \tag{S2d}$$

yielding the following equilibrium values:

$$\hat{S} = \frac{b(\delta + \omega)(\gamma + \delta + \mu)(\gamma + \delta + v)}{\delta(\gamma + \delta + \mu)(\gamma(\delta + \lambda + \omega) + (\delta + \lambda)(\delta + \omega)) + v(\gamma\delta(\delta + \lambda + \omega) + (\delta + \lambda)(\delta + \mu)(\delta + \omega))} \tag{S3a}$$
$$\hat{I} = -\frac{b\lambda(\delta + \omega)(\gamma + \delta + v)}{\gamma\lambda\mu\omega - (\gamma + \delta + v)(\gamma\delta(\delta + \lambda + \omega) + (\delta + \lambda)(\delta + \mu)(\delta + \omega))} \tag{S3b}$$
$$\hat{M} = \frac{b\lambda\mu(\delta + \omega)}{\delta(\gamma + \delta + \mu)(\gamma(\delta + \lambda + \omega) + (\delta + \lambda)(\delta + \omega)) + v(\gamma\delta(\delta + \lambda + \omega) + (\delta + \lambda)(\delta + \mu)(\delta + \omega))} \tag{S3c}$$
$$\hat{R} = \frac{b\gamma\lambda(\gamma + \delta + \mu + v)}{\delta(\gamma + \delta + \mu)(\gamma(\delta + \lambda + \omega) + (\delta + \lambda)(\delta + \omega)) + v(\gamma\delta(\delta + \lambda + \omega) + (\delta + \lambda)(\delta + \mu)(\delta + \omega))} \tag{S3d}$$

Calculating the average age at infection

Because we assume the force of spillover is independent of age, individuals of all ages experience an identical force of infection. As a consequence, the average age at infection is equal to the average age of individuals in the susceptible class, S. To calculate the average age of susceptible individuals, we begin by solving the system of differential equations (S1) under the assumption that disease dependent mortality is negligible ($v = 0$). This yields the following solution for $S(a)$:

$$S(a) = \left(\frac{b e^{-\frac{1}{2}a\left(\gamma+2\delta+\lambda+\sqrt{k_2}+\omega\right)}}{2\sqrt{k_1}(\gamma(\lambda+\omega)+\lambda\omega)}\right)\left(\lambda\left(1-e^{a\sqrt{k_1}}\right)\left(-\gamma^2+\gamma\lambda-\omega(\omega-\lambda)\right)+\right.$$
$$\left.\sqrt{k_1}\left(2\gamma\omega e^{\frac{1}{2}a\left(\gamma+\lambda+\sqrt{k_2}+\omega\right)}+\lambda\left(e^{a\sqrt{k_1}}+1\right)(\gamma+\omega)\right)\right) \tag{S4}$$

where $k_1 = \gamma^2 - 2\gamma(\lambda+\omega) + (\lambda-\omega)^2$ and $k_2 = -2\omega(\gamma+\lambda) + (\gamma-\lambda)^2 + \omega^2$.

Next, we divide this solution for $S(a)$ by the total number of susceptible individuals at steady state (S3a) under the assumption that disease-dependent mortality is negligible ($v = 0$). This gives the probability that a susceptible individual is of age a. Finally, we multiply this by a and integrate over a to calculate the average age of the susceptible population:

$$\bar{\mathcal{A}} = \int_0^\infty a \frac{S(a)}{\hat{S}} \, da \tag{S5}$$

Assuming disease dependent mortality, v, is negligible, the integral in (S5) can be evaluated to yield the following expression for the average age of infection:

$$\bar{\mathcal{A}} = \frac{\delta^2(\gamma+\delta)^2 + \omega^2\left((\gamma+\delta)^2+\gamma\lambda\right) + \omega\left(\gamma\lambda(\gamma+2\delta)+2\delta(\gamma+\delta)^2\right)}{\delta(\gamma+\delta)(\delta+\omega)(\gamma(\delta+\lambda+\omega)+(\delta+\lambda)(\delta+\omega))} \tag{S6}$$

Approximating the burden of disease with lifelong immunity

If we assume that the rate at which infected individuals become clinically diseased, μ, is small relative to the rate at which individuals recover from infection, γ, the number of individuals in the M class will be negligible and can be ignored to a first approximation. Thus, the approximation we pursue here is directly

applicable to only those infectious diseases that generally produce mild symptoms and rarely proceed to clinical disease. Combining this assumption with the assumption that immunity is lifelong allows the steady state of the system of equations (1) to be approximated by:

$$\frac{dS}{da} = -\lambda S - \delta S \tag{S7a}$$

$$\frac{dI}{da} = \lambda S - \gamma I - \delta I \tag{S7b}$$

$$\frac{dR}{da} = \gamma I - \delta R \tag{S7c}$$

Solving this system of equations with initial conditions $S(0) = b$, $I(0) = 0$, and $R(0) = 0$ yields the following steady state solution for the number of infected individuals of age a:

$$\hat{I} = -\frac{b\lambda\left(e^{-a(\gamma+\delta)} - e^{-a(\delta+\lambda)}\right)}{\gamma - \lambda} \tag{S8}$$

Next, we assume that, in addition to being small, the rate of progression to clinical disease is linearly related to age:

$$\mu = \mu_0 + \alpha a \tag{S9}$$

where α must be sufficiently small for μ to remain small over all age classes.

Substituting (S8) and (S9) into equation (3) and carrying out the integration yields the following solution:

$$\hat{\mathcal{B}} = \frac{\delta\lambda(\alpha(\gamma + 2\delta + \lambda) + \mu_0(\gamma + \delta)(\delta + \lambda))}{(\gamma + \delta)^2(\delta + \lambda)^2} \tag{S10}$$

This expression has an internal maximum with respect to λ ($\mathcal{B}' = 0$ and $\mathcal{B}'' < 0$) anytime the following condition holds:

$$\mu_0 < \frac{\alpha\gamma}{\delta(\gamma + \delta)} \tag{S11}$$

Solving this expression for α yields expression (9) of the main text.

Approximating the burden of disease with waning immunity

We make the same assumptions and follow the same sequence of steps used in the previous section. Assuming infection rarely proceeds to clinical disease, the equilibrium state of the system is approximated by the following system of ordinary differential equations:

$$\frac{dS}{da} = -\lambda S + \omega R - \delta S \tag{S12a}$$

$$\frac{dI}{da} = \lambda S - \gamma I - \delta I \tag{S12b}$$

$$\frac{dR}{da} = \gamma I - \omega R - \delta R \tag{S12c}$$

Solving this system shows that, at equilibrium, the number of infected individuals of age, a, is given by:

$$\hat{I} = \frac{b\lambda\left(\left(e^{a\sqrt{k_1}}-1\right)(\gamma(2\lambda+\omega)+\omega(\lambda-\omega)) - \sqrt{k_1}\omega\left(-2e^{\frac{1}{2}a\left(\gamma+\lambda+\sqrt{k_2}+\omega\right)}+e^{a\sqrt{k_1}}+1\right)\right)e^{-\frac{1}{2}a\left(\gamma+2\delta+\lambda+\sqrt{k_2}+\omega\right)}}{2\sqrt{k_1}(\gamma(\lambda+\omega)+\lambda\omega)} \tag{S13}$$

where the compound constants k_1 and k_2 are introduced only temporarily so that the expression can be displayed on a single line. These compound constants take values:

$$k_1 = \gamma^2 - 2\gamma(\lambda+\omega) + (\lambda-\omega)^2 \tag{S14a}$$

$$k_2 = -2\omega(\gamma+\lambda) + (\gamma-\lambda)^2 + \omega^2 \tag{S14b}$$

Substituting (S13) and (S9) into equation (3) and carrying out the integration yields the following solution:

$$\hat{\mathcal{B}} = \frac{\delta\lambda\left(\alpha\left(\gamma\left(\delta^2+2\delta\omega+\omega(\lambda+\omega)\right)+(2\delta+\lambda)(\delta+\omega)^2\right)+\delta\mu_0(\delta+\omega)(\gamma(\delta+\lambda+\omega)+(\delta+\lambda)(\delta+\omega))\right)}{(\gamma\delta(\delta+\lambda+\omega)+\delta(\delta+\lambda)(\delta+\omega))^2} \tag{S15}$$

To identify the conditions required for negative impacts of spillover reduction to arise, we again proceed by finding conditions where $\mathcal{B}' = 0$ and $\mathcal{B}'' < 0$. The first condition is satisfied for values of $\lambda > 0$ anytime:

$$\alpha > \frac{\delta\mu_0(\gamma+\delta)(\delta+\omega)(\gamma+\delta+\omega)}{\gamma\left(-\omega(\gamma+2\delta)+\delta(\gamma+\delta)-\omega^2\right)} \tag{S16}$$

and either inequality (S17) or inequalities (S18) are satisfied:

$$\delta \geq \omega(1+\sqrt{2}) \tag{S17}$$

$$\omega < \delta < \omega(1+\sqrt{2}) \tag{S18a}$$

$$\delta > \omega + \frac{\sqrt{\gamma^2 + 8\omega^2} - \gamma}{2} \tag{S18b}$$

The second condition, $\mathcal{B}'' < 0$, is guaranteed to be true if $\mathcal{B}' = 0$. Together, these conditions demonstrate that waning immunity makes negative consequences of spillover reduction much less likely.

Extension to an alternative public health metric: expected lifespan

Using a sequence of operations similar to those used to study burden with lifelong immunity, we sought an approximation for the expected lifespan of an individual as a function of the force of infection. Equations (S1) with initial conditions $S(0) = 1$, $I(0) = 0$, $M(0) = 0$, and $R(0) = 0$ can be interpreted as the probability that an individual is alive and in each of the respective immunological classes. The probability that an individual is alive at age a (the survival function) is thus $x = S + I + M + R$ and obeys the differential equation

$$\frac{dx}{da} = -\delta x - vM. \tag{S19}$$

Assuming lifelong immunity so $\omega = 0$, linearly increasing rate of progression to clinical disease (equation (S9)), and that M can be taken to quasi-steady state in equations (S1),

$$M \approx \frac{(\mu_0 + \alpha a)I}{v + \gamma + \delta}. \tag{S20}$$

Substituting our approximation for the number of infected individuals of age a (equation (S8)) into (S20) and the resulting equation into (S19) and solving the differential equation yields

$$\begin{aligned} x(a) =& \frac{ve^{-a(\gamma+\delta+\lambda)}\left(\gamma^2 e^{a\gamma}(a\alpha\lambda + \alpha + \lambda\mu_0) - \lambda^2 e^{a\lambda}(a\alpha\gamma + \alpha + \gamma\mu_0)\right)}{\gamma^2\lambda(\gamma-\lambda)(\gamma+\delta+v)} + \\ & \frac{e^{-a\delta}\left(\gamma^2\lambda(\gamma+\delta) - v(\alpha(\gamma+\lambda) + \gamma\lambda(\mu_0 - \gamma))\right)}{\gamma^2\lambda(\gamma+\delta+v)} \end{aligned} \tag{S21}$$

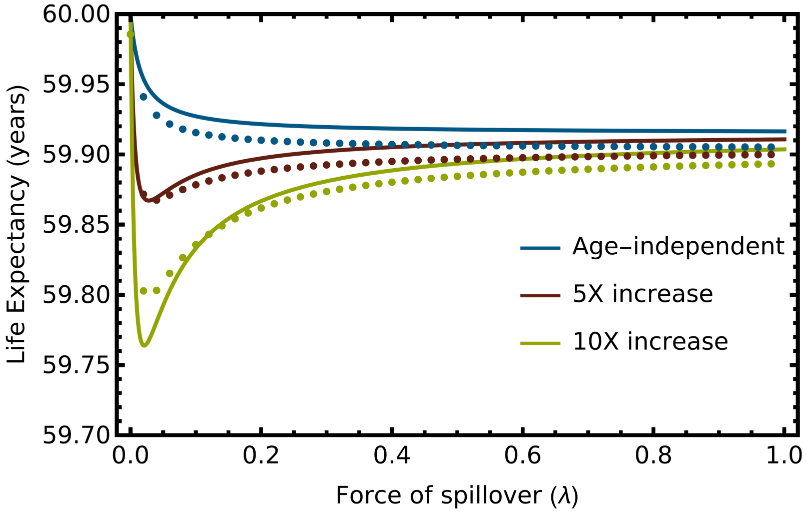

Figure 1: Life expectancy in years as a function of the force of spillover (λ) for disease severity that increases at different rates with advancing age (colors). Here, $\mu_0 = 1$ and the slope of the rate at which individuals transition to severe disease was set to be independent of age (blue line; $\alpha = 0$), increase 5-fold (red line; $\alpha = 1/15$), or increase 10-fold (yellow line; $\alpha = 3/20$) from the time of birth to age at which individual reaches their expected natural lifespan ($1/\delta$). Dots are results of numerical solutions to the exact model (equations (S1)) and lines are approximations (equation (S22)). The remaining parameters were $b = 1$, $\delta = 1/60$, $v = 1$, $\gamma = 365/14$, and $\omega = 0$.

Integrating this equation yields an approximation for life expectancy:

$$\int_0^\infty x(a)da = \frac{\gamma+\delta}{\delta(\gamma+\delta+v)} + \frac{v\left(-\alpha\gamma\lambda - 2\alpha\delta\lambda - \alpha\lambda^2 + \gamma^2(\delta+\lambda)^2 + \gamma(\delta+\lambda)(2\delta(\delta+\lambda) - \lambda\mu o) + \delta^4 + 2\delta^3\lambda + \delta^2\lambda^2 - \delta\lambda\mu o(\delta+\lambda)\right)}{\delta(\gamma+\delta)^2(\delta+\lambda)^2(\gamma+\delta+v)} \tag{S22}$$

Conditions for this equation to have an internal minimum with respect to λ are identical for those for burden, equation (9) in the main text. In Figure 1, we compared this analytical approximation for life expectancy as a function of the force of spillover to numerical solutions of the exact model that does not assume that the rate of transition to clinical disease is rare (Figure 1)

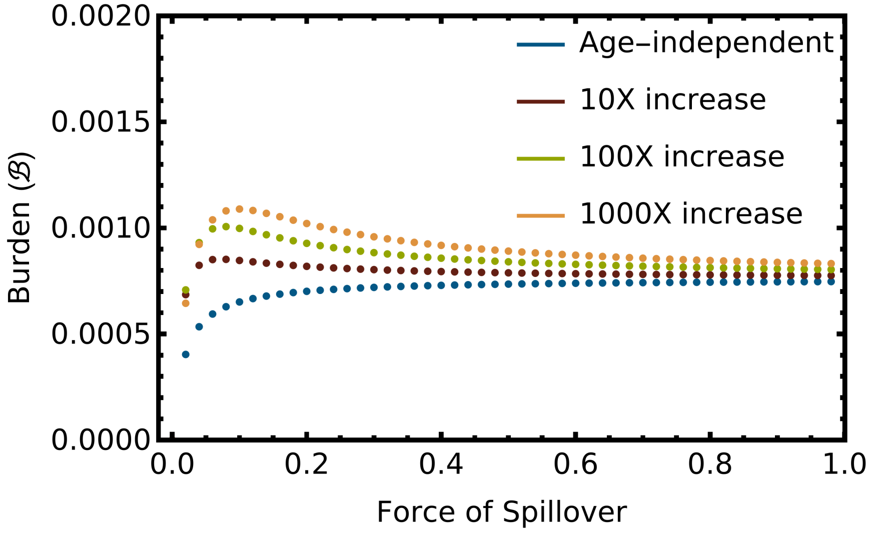

Figure 2: Numerical solutions for the health burden of a zoonotic disease, $\mathcal{B}$, as a function of the force of spillover (λ) for disease severity that increases exponentially with advancing age. The rate at which infected individuals become diseased increases following equation (S23) with intercept set to $\mu_0 = 1$ and slope set such that the rate of transition to disease is independent of age (blue line; $\alpha = 0$), increases 10-fold (red line; $\alpha = 0.038$), increases 100-fold (green line; $\alpha = 0.077$), or increases 1000-fold (orange line; $\alpha = 0.115$) from the time of birth to the age at which an individual reaches their expected natural lifespan ($1/\delta$). The remaining parameters were $b = 100$, $\gamma = 365/14$, $\delta = 1/60$, $v = 1$, and $\omega = 0$.

Extension to non-linear relationship between age and disease severity

We explored the impact of an exponentially increasing rate of progression to clinical disease with age:

$$\mu = \mu_0 e^{\alpha a}. \tag{S23}$$

Figure 2 shows the clinical burden of zoonotic disease at steady state (Equation 3) found from numerically solving equations (S1) with equation (S23). As with our results for a linearly increasing rate of progression to clinical disease with age, we found that burden is maximized for intermediate levels of spillover provided that the rate of progression to clinical disease increases sufficiently rapidly with age.

Application to Lassa virus

Summary of parameter estimates for Lassa virus

The parameter estimates used for analyses of Lassa virus are reported in Table 1

Parameter	Biological interpretation	Estimate	Reference
δ	Rate of disease-independent mortality	0.0165	[1]
μ	Rate of progression to clinical disease	3.05	[2]
v	Rate of disease-dependent mortality	5.22	[2]
γ	Rate of recovery from infection	12.17	[3]
ω	Rate at which immunity is lost	0.064	[4]

Table 1: Model parameters, biological interpretations, estimated values for the Lassa virus system, and references. The rate of progression to clinical disease was calculated based on a probability of developing symptoms over the course of infection equal to 20%. The mortality rate, v, in cases where symptoms develop, was calculated based on a case fatality rate of 30%. All rates are in years.

Quantifying the relationship between age and transition to clinical disease

To fit our models to data from the Lassa virus system, we must estimate the parameters μ_0 and α that define how the rate of advance to clinical disease depends on age at infection. To a reasonable approximation, we can use data provided by [5] that reports the proportion of Lassa virus infected patients admitted to three hospitals in Nigeria that ultimately succumbed to infection as a function of age. This data provides support for a linear increase in disease severity with advancing age (Figure 3). To use this data in our model, we transformed case fatality rate (proportion admitted who ultimately succumbed) to the rate at which individuals advance from infection to clinical disease, μ, using equation (4). We then fit the data to the linear relationship described by equation (8) using least squares to yield estimated values of $\mu_0 = 1.26$ and $\alpha = 0.091$.

Estimating the force of spillover from seroprevalence estimates

The equilibrium solutions (S3) can be used to estimate the force of spillover for populations where the seroprevalence of Lassa virus antibodies is known. Specifically, at equilibrium, the proportion of the population in the R class (proportion seropositive) is given by:

$$\mathcal{R} = \frac{\hat{R}}{\hat{S}+\hat{I}+\hat{M}+\hat{R}} = \frac{\gamma\lambda(\gamma+\delta+\mu+v)}{(\gamma+\delta+\mu)(\gamma(\delta+\lambda+\omega)+(\delta+\lambda)(\delta+\omega))+v(\gamma(\delta+\lambda+\omega)+(\delta+\omega)(\delta+\lambda+\mu))} \tag{S24}$$

Solving equation (S24) for the force of spillover, λ, yields the estimator reported in the main text as equation (5).

Sensitivity to relationship between age and transition to clinical disease

We evaluated how sensitive our conclusions were to the precise linear relationship between disease severity and advancing age that we estimated using data in [5] by studying scenarios where disease severity increased more rapidly with advancing age. Specifically, we considered cases where the slope, α, increased 1.5 times and 2.0 times as rapidly as we estimate from the data. The results of this analysis demonstrate

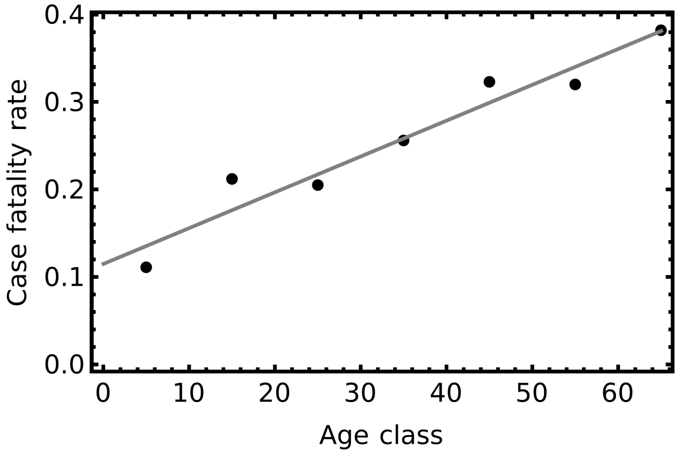

Figure 3: The proportion of individuals with laboratory confirmed Lassa virus infections admitted to three hospitals in Nigeria between January 1, 2018 and May 6, 2018 that ultimately succumbed to the infection as a function of age [5]. The black line shows a linear fit to the data based on least squares.

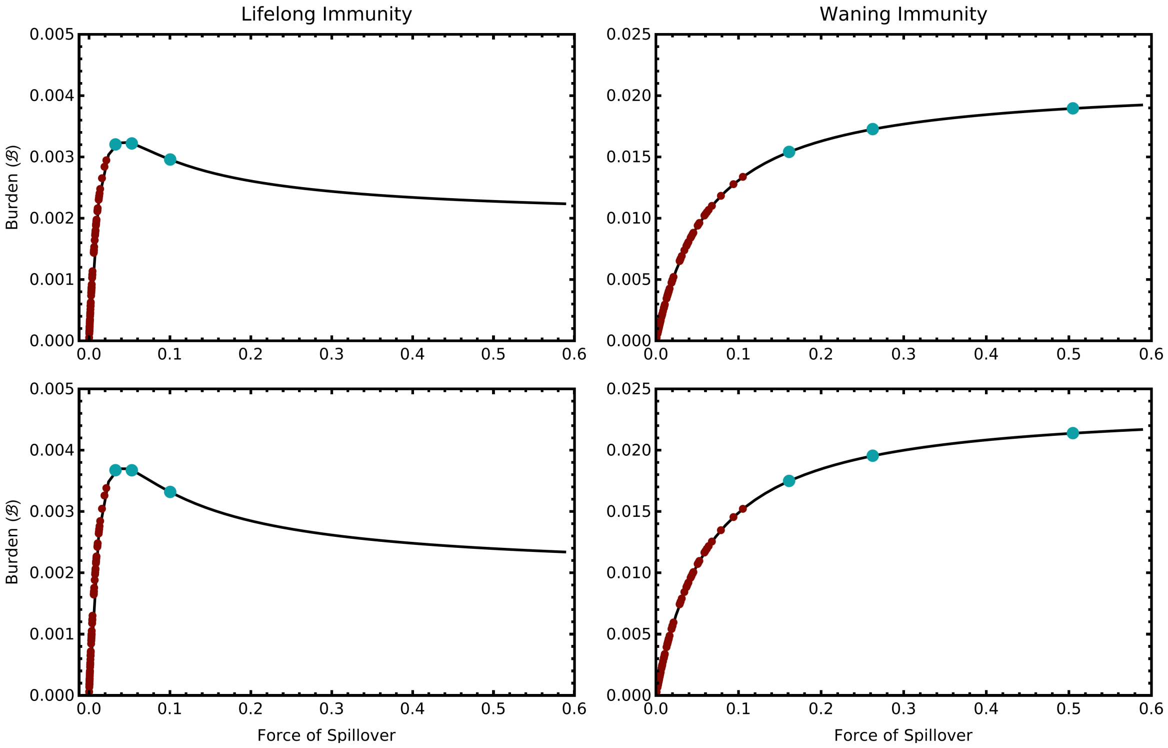

Figure 4: The sensitivity of estimates for the burden of Lassa virus infection to the estimated value of the slope of the line (α) relating age at infection to the rate of transition to clinical disease. The first row shows a scenario where the value of α exceeds the value we estimate from the data in [5] by 150% and the second row a scenario where it exceeds the estimated value by 200%. The left hand column shows the results when immunity is assumed to be lifelong and the right hand column the results when immunity wanes. The black line is the theoretical prediction for each case as a function of the force of spillover. The red dots show the force of spillover estimated for actual sites in West Africa where systematic serosurveys have been conducted. The blue dots are hypothetical populations with seroprevalances equal to $\mathcal{R} = 0.65$, $\mathcal{R} = 0.75$, and $\mathcal{R} = 0.85$. For the case of waning immunity, the expected duration of immunity is set to 15.63 years ($\omega = 0.064$) as estimated by [4]. Birth rate of the human population was set to $b = 24.75$ which yields a local population size of 1500. Other parameter values were as described in Table (1). Note that the burden of zoonotic disease is greater with waning immunity because reinfection is possible.

that our general conclusions remain robust – spillover reduction is not expected to negatively impact human health in any populations within our data set. However, the results do show that – if negative consequences were to occur – their magnitude could be considerably greater (Figure 4).

References

[1] WorldBank. Life epectancy at birth, total (years).; 2022. Available from: https://data.worldbank.org/indicator/SP.DYN.LE00.IN.

[2] Garry RF. Lassa fever — the road ahead. Nat Rev Microbiol. 2023;21(2):87–96. doi:10.1038/s41579-022-00789-8.

[3] Basinski AJ, Fichet-Calvet E, Sjodin AR, Varrelman TJ, Remien CH, Layman NC, et al. Bridging the gap: Using reservoir ecology and human serosurveys to estimate Lassa virus spillover in West Africa. PLoS Comput Biol. 2021;17(3):e1008811. doi:10.1371/journal.pcbi.1008811.

[4] Mccormick J, Webb P, Krebs J, Johnson K, Smith E. A prospective-study of the epidemiology and ecology of Lassa fever. Journal of Infectious Diseases. 1987;155(3):437–444. doi:10.1093/infdis/155.3.437.

[5] Ilori EA, Furuse Y, Ipadeola OB, Dan-Nwafor CC, Abubakar A, Womi-Eteng OE, et al. Epidemiologic and Clinical Features of Lassa Fever Outbreak in Nigeria, January 1–May 6, 2018. Emerg Infect Dis. 2019;25(6):1066–1074. doi:10.3201/eid2506.181035.
