# Supplementary material for: Quantifying the risk of spillover reduction programs for human health
Source: PLoS Comput Biol. 2024 Aug 15;20(8):e1012358. doi: 10.1371/journal.pcbi.1012358 (PMC11349207; doi:10.1371/journal.pcbi.1012358)
Supplement: Attachment — Submitted filename: Nuismer_Response.pdf [file pcbi.1012358.s002.pdf]

20 June 2024

Dear Editors,

We would like to submit our revised article, "Quantifying the risk of spillover reduction programs for human health" for potential publication in *PLoS Computational Biology.* We appreciated the positive response to our manuscript and found the suggestions made by the editor and reviewers to be very helpful. Below, we explain (in **bold**) how we have addressed each specific point (in *italic*).

Editor

Reviewers emphasized the need for a more nuanced characterization of the conditions under which spillover translates to negative public health consequences in real-world scenarios. Both reviewers offered valuable suggestions for strengthening this aspect, proposing ideas to explore further aspects linking theoretical concepts and practical applications.

We appreciate this perspective and now provide a more nuanced interpretation along with additional supporting analyses. Specifically, we now emphasize that the conditions required for spillover reduction to have negative public health impacts are quite restrictive. In addition, we analyze non-linear relationships between age and disease severity and alternative metrics for the public health impacts of spillover. The specific changes we have made are detailed in our responses to the individual reviewers which follow.

Reviewer 1

One of my primary concerns is that the manuscript currently leaves the reader with the message that spillover reduction is something that should be approached with caution, but the results don't seem to align with this perspective. It is not clear how often reduction of spillover will actually result in negative public health consequences in practice, despite being presented as a significant risk. Perhaps the authors could point to examples of real systems where these negative consequences may be likely, or alternatively ease up on the message that reducing spillover can be bad – I would probably suggest the latter given that the authors have already shown that even in the worst cases, reducing spillover leads to short term benefits (akin to vaccine honeymoon periods).

We agree and have reduced the emphasis on possible negative consequences of spillover reduction which our results suggests are very unlikely. Instead we adopt a more balanced presentation that explains the possible risks but emphasizes their likely rarity. These changes are made in the abstract, introduction, and discussion (e.g., lines 23-26, lines 59-61, lines 271-291, lines 343-356). We also increase the focus on the more likely outcome where initial benefits of spillover reduction fade over time (e.g., lines 23-26 and lines 279-282).

Furthermore, observing negative public health consequences could depend on the metric used to measure public health burden. The authors currently use the number of severe disease cases. While this is reasonable, other metrics seem more relevant to me. For example, does reducing spillover also reduce life expectancy or increase DALYs (disability adjusted life years)? As we learned from COVID, the health of elderly individuals is not always valued in the same way as the health of children/workers.

This is also a good point. To investigate if the results differed for alternative metrics of public health burden, we studied life expectancy as the reviewer suggests. We found that the conditions required for spillover reduction to reduce expected lifespan are identical to those required for spillover reduction to increase severe disease. These results are now reported in a new section of the Appendix and a new figure (Appendix Figure A1). To further explore consequences of spillover reduction for life expectancy, we also calculated how life expectancy

changes over the course of a spillover-reducing intervention. These results are now reported in the new figures Figures 4-6 which replace the original Figures 4-5.

It was shown that disease burden could be maximized for intermediate spillover only if the relationship between age and severity was sufficiently strong. Since this suggests that burden would be driven by infections of older individuals in the population, it might be useful to show the distribution of ages at infection rather than just the average age of infection in Figs 4 and 5. This would probably become even more important if risk increased non-linearly with age, as it likely does, which could be worth discussing.

Although we appreciate this suggestion and agree showing the entire distribution would provide more information, we could find no simple and efficient way to display this information within the figure and still convey the important temporal trends. This challenge was magnified by adding a fourth panel to these figures to display the average human lifespan.

Reducing spillover seemingly reduces overall infection prevalence (percent infected, as shown in figures 4, 5) regardless of the impact on the metric of disease burden. The model does not currently incorporate any human-to-human transmission. Perhaps the authors could speculate on how limited human transmission (i.e., $R_0 < 1$) might impact the change in public health burden as spillover is reduced, so that this theory could be applied more generally.

We now include a more detailed discussion of how weak human-to-human transmission might alter the results (Lines 322-327). We speculate that limited human-to-human transmission ($R_0 < 1$) would have no significant impact on our results.

The ecological driving mechanisms of spillover can result in inhomogeneous exposure over time and between different age groups, and this could have significant implications on the metric for disease burden. Namely, if exposure risk and disease severity are inversely related (e.g. children more likely to come in contact with wildlife), reducing spillover would be unlikely to increase burden since high-risk individuals are unlikely to be exposed regardless. It would be useful to at least consider how age-dependencies in exposure risk and disease severity might interact within this framework.

This is an excellent point which we now expand on in the discussion (lines 327-333).

Is the maximal burden for intermediate levels of spillover only possible with linear relationships, or would this trend hold for any function that is sufficiently quickly increasing. Could other shapes possibly yield a similar trend (i.e., high risk in very young and very old individuals)?

This is a good point. Although we could not find general solutions for functions of arbitrary form, we explored an additional case where the relationship between severity and age increased exponentially. The effect is still possible with this non-linear relationship (Appendix figure A2.

The parameter choice for Figure 3 seemed strange: Why were these particular values used when they hardly show increased burden for intermediate spillover?

This is a good point. We now use parameters for this figure that yield the largest effect shown in Figure 2.

In Figures 4 and 5, perhaps consider adding mean lines from several simulations instead of just points from a single replicate. This could help demonstrate consistency between simulation replicates. It does not seem relevant in this particular context, but this does not necessarily show if some simulations reach different equilibria under the same parameter set.

This is a good suggestion. We have replaced Figures 4 and 5 with Figures 4-6 to convey more information. These revised figures also now show the results from ten independent simulation runs.

I was curious about the observation that basically any waning removed the observed pattern. Is this because of the assumption that waning is exponential. Would simulations with discrete waning periods or multiple recovered classes (yielding gamma distributed wait times) "recover" this pattern so to speak?

This is an interesting question for which we do not have a clear answer. It may be that the impact of waning is magnified through the exponential assumption. Although we could investigate this by developing and analyzing additional models with discrete waning periods, we do not believe this would fundamentally change the results although it might reduce the moderating influence of waning immunity.

Typo in Figure 2 key: "Age-independent"

We have corrected the typo.

I really don't like the term "exact numerical solution" in the Figure 2 legend. I suppose there are some cases where a numerical solution can be exact, but is that the case here? Perhaps "numerical solution to the exact model" would be more accurate.

We agree and have made the suggested change. For consistency, we also made this change to the legend of Figure 3 where the same text appeared.

Reviewer 2

Lines 57-59: "Our models apply to those zoonotic diseases caused primarily by repeated, direct spillover from an animal reservoir rather than an initial spillover followed by sustained human-to-human transmission." It would be interesting if the authors briefly discussed in their conclusion section the assumption of a sub-critical pathogen throughout the paper. It does not seem that the main theoretical argument depends on this assumption and the super-critical case could be an interesting topic for a sequel paper.

We now discuss this assumption of our model in greater detail and speculate on the consequences of including weak human-to-human transmission (lines 324-327).

Lines 134-138 (and appendix): "Assuming the population is at a steady state..." This assumption is used in the estimation, as well as in different parts of the paper. Does this assumption hold in the data? It should not be hard to check whether the population under study experiences population growth and/or whether the demographic characteristics are changing over the sample period.

This is a good point that we cannot, unfortunately, address. The data we have available to us comes from cross-sectional serosurveys of human populations. There is, as far as we know, no simple methodology that would allow us to test if these populations are at steady state. Strictly speaking, it is unlikely that these populations are at a demographic steady state. It is also unlikely that these populations are at an epidemiological steady state due to changes in the population abundance of the reservoir animals and its putative displacement by invading black rats. We now address this important assumption and its possible consequences in the discussion (Lines 333-338).

Line 146: "we calculate the average age at infection for our model" Related to the above equation, does the theoretical average infection age match the infection pattern observed in the data? It might be useful to comment on this since, as shown later in paper, the steady state assumption is not entirely innocuous.

Although we agree that this would be interesting to explore, we do not, in general, have access to the age-structured human seroprevalence data required to assess this. An additional complication is that because we estimate the force of infection from the human seroprevalence data, the average age at infection would match that in the data by reason of circularity.

Thank you again for the encouraging feedback on our manuscript and the helpful suggestions for improvement. We hope that our changes have completely addressed the issues and that our manuscript is now suitable for publication in *PLoS Computational Biology.* If any additional changes are required, please let us know.

Sincerely,

Scott L. Nuismer
